# Fatty Acid Lingual Application Activates Gustatory and Reward Brain Circuits in the Mouse

**DOI:** 10.3390/nu10091246

**Published:** 2018-09-06

**Authors:** Yvan Peterschmitt, Souleymane Abdoul-Azize, Babar Murtaza, Marie Barbier, Amira Sayed Khan, Jean-Louis Millot, Naim Akhtar Khan

**Affiliations:** 1 Neurosciences Intégratives et Cliniques EA481, Université de Bourgogne Franche-Comté (UBFC), 25000 Besançon, France; yvan.peterschmitt@univ-fcomte.fr (Y.P.); marie.barbier03@edu.univ-fcomte.fr (M.B.); 2 Unité Inserm U1234, Université de Rouen/IRIB, Faculté de Médecine et Pharmacie, 76183 Rouen CEDEX, France; souleymane.abdoul-azize@univ-rouen.fr; 3 Physiologie de la Nutrition & Toxicologie (NUTox), Agro-Sup, UMR U1231 INSERM/Université de Bourgogne Franche-Comté (UBFC), 6, Boulevard Gabriel, 21000 Dijon, France; babarmurtaza87@gmail.com (B.M.); amira.khan@u-bourgogne.fr (A.S.K.)

**Keywords:** linoleic acid, gustation, hedonic, BDNF, fat taste, c-Fos, Zif-268, Glut-1

## Abstract

The origin of spontaneous preference for dietary lipids in humans and rodents is debated, though recent compelling evidence has shown the existence of fat taste that might be considered a sixth taste quality. We investigated the implication of gustatory and reward brain circuits, triggered by linoleic acid (LA), a long-chain fatty acid. The LA was applied onto the circumvallate papillae for 30 min in conscious C57BL/6J mice, and neuronal activation was assessed using c-Fos immunohistochemistry. By using real-time reverse transcription polymerase chain reaction (RT-qPCR), we also studied the expression of mRNA encoding brain-derived neurotrophic factor (BDNF), Zif-268, and Glut-1 in some brain areas of these animals. LA induced a significant increase in c-Fos expression in the nucleus of solitary tract (NST), parabrachial nucleus (PBN), and ventroposterior medialis parvocellularis (VPMPC) of the thalamus, which are the regions known to be activated by gustatory signals. LA also triggered c-Fos expression in the central amygdala and ventral tegmental area (VTA), involved in food reward, in conjunction with emotional traits. Interestingly, we noticed a high expression of BDNF, Zif-268, and Glut-1 mRNA in the arcuate nucleus (Arc) and hippocampus (Hipp), where neuronal activation leads to memory formation. Our study demonstrates that oral lipid taste perception might trigger the activation of canonical gustatory and reward pathways.

## 1. Introduction

Taste modality serves as an important factor for food choice and for appreciating its hedonic value [1]. There are five basic taste qualities known hitherto in rodents and humans: sweet, sour, bitter, salty, and umami [2]. The specific receptors and cells for each of the five basic taste modalities have been identified and characterized [3]. The series of events that occur before and after the ingestion of food, leading to taste perception and preference, are a topic of wide interest. Recently, convincing evidence has started to accumulate in favor of fat as the sixth fat taste quality in rodents and humans [4]. The two principal receptors of fat taste, CD36 and GPR120, have finally been identified in human taste bud cells, and their sensitivity to fatty acid stimuli has been shown to be altered in obesity [5]. The cellular and molecular mechanisms of fat taste perception have recently been elucidated [4,6].

There are a few studies that have shed light on fat-eating behavior and brain activity. Our laboratory has demonstrated that the addition of a fatty acid on mouse tongues induced the expression of c-Fos immunoreactivity in the nucleus of the solitary tract (NST), the first gustatory relay in the brain [7]. We did not address the question of whether other parts of the brain are also activated during this experimental approach, though several investigators, by employing different methods, have concluded that the primary taste cortex, orbitofrontal cortex, and amygdala are activated by the perception of dietary lipids [8,9,10,11]. Tzieropoulos et al. (2013) reported that dietary fat was able to induce sustained reward response in human brains. Eldeghaidy et al. [12] used functional magnetic resonance imaging (fMRI) in human subjects, and suggested that taste, appetite, and reward-related brain areas were responsive to nutritional status and received sensory and interoceptive signals of motivation and hedonic value in response to a fat-rich diet. Other fMRI studies in humans have also demonstrated that administration of dietary lipids activates cerebral taste, texture, and reward areas [13,14,15,16].

The abovementioned studies show that different brain areas might be activated by dietary fat; however, there is a dearth of information on the identification of sequential activation of cerebral areas/pathways that are activated in response to taste bud stimulation by dietary fat prior to ingestion of the bolus. Information on this subject would be crucial not only to better understand the fundamental mechanisms of fat intake and its related addiction, but also to modulate fat-eating behavior that is altered in obese subjects [17]. Keeping this argument in view, we designed the present study wherein we added linoleic acid (LA), a long-chain fatty acid, on the circumvallate papillae, which are rich in fat taste receptors, of conscious mice and assessed the neural activity using immunocytochemical localization of c-Fos protein in different brain areas. We also analyzed the mRNA expression of brain-derived neurotrophic factor (BDNF), involved in synaptic plasticity and memory processes, Zif-268, an immediate-early gene, and Glut-1, another marker of neuronal activation during enhanced glucose demand in three brain areas of these animals [18].

## 2. Materials and Methods

### 2.1. Animals and Experimental Set-Up

Experiments were carried out on 6–10-week-old C57BL/6J male mice (Janvier, Le Genest-St-Isle, Mayenne, France). Animals were group-housed under standard laboratory conditions (12 h:12 h, light/dark cycle; 22 ± 2 °C, 50–60% humidity) and fed with standard pelleted food (Scientific Animal Food & Engineering, Augy, France) and water ad libitum. All experiments were designed to minimize animal suffering and the number of animals. The protocols were approved by the regional ethical committee of Burgundy University in compliance with European guidelines for the use and care of laboratory animals.

In the first set of experiments, the neuronal activation along the canonical gustatory pathway was systematically assessed after oral lipid stimulation using c-Fos immunohistochemistry. In order to avoid any stressful situation, the mice were accustomed to gentle handling for 5 days in order to apply the fatty acid. On the 6th day, they were gently handled similarly and linoleic acid (LA, 18:2 n-6) (Sigma-Aldrich, St. Louis, MO, USA) at 50 µM in 70 µl (*w*/*v*) was slowly placed with the help of a spatula for 30 min on the circumvallate papillae. Xanthan gum (0.3% *w*/*v*, Sigma-Aldrich USA), which mimics lipid texture, was similarly applied onto the circumvallate papillae of control animals. At the end of oral stimulation, the animals were injected, intraperitoneally, with sodium pentobarbital (40 mg/kg), the thoracic cavity was opened, and mice were perfused intracardially with 50 mL of ice-cold saline (NaCl, 0.9%), followed by 50 ml of ice-cold 4% paraformaldehyde in 0.1 M phosphate buffer, pH 7.4. The entire brain was removed and post-fixed by incubation in 4% paraformaldehyde for 2 h. Samples were cryoprotected by overnight incubation in 30% sucrose in 0.1 M phosphate buffer. Brain samples were embedded in a Tissue-Tek^®^OCT compound (Sakura FineTek, Torrance, CA, USA), frozen on dry ice, and stored at −20 °C before c-Fos immunohistochemical processing.

In the second set of experiments, using real-time reverse transcription polymerase chain reaction (RT-qPCR), we investigated the downstream molecular pathways/candidates in NTS, arcuate nucleus, and hippocampus at mRNA level. The total RNA from different brain areas was isolated by Trizol Reagent (Invitrogen, Thermo Fisher Scientific, Waltham, MA, USA). The quality of isolated RNA was determined using denaturing agarose gel electrophoresis. Then, the RNA was quantified by determining its UV absorbance at 260 nm. Five hundred nanograms of total RNA was reverse-transcribed with an iScript cDNA synthesis kit according to the manufacturer’s instructions (Bio-Rad, Berkeley, CA, USA). RT-PCR was performed on the iCycler iQ real-time detection system, and amplification was undertaken using SYBR Green detection. Primers against the genes of interest were as follow: BDNF (forward 5′-TTGGATGCCGCAAACATGTC-3′; reverse, 5′-CTGCCGCTGTGACCCACTC-3′), Zif-268 (forward, 5′-GCGAACAACCCTATGAGC-3′; reverse, 5′-GGTCGGAGGATTGGTC-3′), Glut-1 (forward, 5′-GCTGTGCTTATGGGCTTCTC-3′; reverse, 5′-CACATACATGGGCACAAAGC-3′). The cycling conditions used were: 95 °C for 10 min; and 40 cycles of 95 °C for 15 s, 60 °C for 1 min. The amplification was carried out in a total volume of 20 µL, which contained 10.0 µL SYBR Green Supermix buffer (50 mMKCl; 20 mMTris-HCl (pH 8.4); 3 mM MgCl_2_; 0.2 mM of each dNTP, 0.63 U iTaq DNA polymerase, and SYBR Green 1.0 nM fluorescein), 0.75 µL (0.3 mM) of each primer, and 1.5 µL diluted cDNA. Results were evaluated using iCycleriQ software, and a relative quantification of mRNA in different groups was determined. The relative amounts of RNA were normalized to the amount of the endogenous control 18S using StepOne software version 2.2-2010 (Applied Biosystems, Thermo Fisher Scientific, Waltham, MA, USA) and the Δ Ct method.

### 2.2. Immunohistochemistry

#### Fos Immunostaining

Fifty μm coronal sections were cut with a cryostat at −20 °C through the NST (nucleus of the solitary tract, bregma −0.48 to −7.08 mm), PBN (parabrachial nucleus, bregma −4.96 to −5.32 mm), VPM (ventral posteromedial thalamic nucleus, bregma −1.58 to −2.46 mm), VPMPC (ventroposterior medialis parvocellularis, bregma −1.94 to −2.30 mm), CeA (central amygdaloid nucleus, bregma −0.94 to −1.82 mm), PSTN/CbN (parasubthalamic nucleus/calbindin nucleus, bregma −2.06 to −2.54 mm), AI (agranular insular cortex, bregma −0.94 to +1.54 mm), GI/DI (granular/dysgranular insular cortex, bregma −0.94 to +1.54 mm), Hipp (hippocampus, bregma −1.22 to −2.18 mm), BLA (basolateral amygdaloid nucleus, bregma −1.70 to +1.82 mm), VTA (ventral tegmental area, bregma −2.92 to +3.88 mm), Acb (accumbens nucleus, bregma +1.70 to +0.98 mm), mPFC (medial prefrontal cortex, bregma +1.98 to +1.54 mm), Arc (arcuate nucleus, −1.46 to −2.46 mm caudal to bregma), and Hbn (habenula, bregma −1.22 to −2.18 mm), according to the atlas of Paxinos & Franklin (2001) [19]. Free-floating sections were then collected in 0.1 M phosphate buffer, pH 7.4, and processed for c-Fos immunohistochemistry. Sections were incubated overnight with rabbit anti-c-Fos (1:20,000, Calbiochem^®^, Paris, France) primary antibodies diluted in 0.1 M phosphate buffer at pH 7.4, containing 0.3% Triton X100 and 3% normal goat serum (*v*/*v*). Subsequently, sections were incubated for 2 h at room temperature with the biotinylated goat antirabbit secondary antibodies (1:4000, Vector laboratories, Burlingame, CA, USA). The formed antigen–antibody complexes were visualized through the avidin–biotin–horseradish peroxidase procedure (Vectastain Elite ABC kit; Vector Laboratories, USA), using 3,3′-diaminobenzidine (0.04%) as the chromogen. Sections were mounted on gelatin-coated slides, dehydrated, and coverslipped with DePeX (VWR International Ltd., Poole, UK) mountant.

### 2.3. Quantification of c-Fos Immunopositive Neurons

Sections were analyzed under a 40× objective of a light-optical microscope (Nikon Eclipse E600, Nikon Instruments Inc., Melville, NY, USA) equipped with a digital camera (Nikon Digital Sight DS-Fi1). c-Fos immunoreactive nuclei were quantified on photomicrographs of the regions of interest (ROI) using the imaging software Image J (National Institutes of Health, Bethesda, MD, USA). A cell was considered as labeled (positive) for c-Fos when the brown-black DAB-staining was unambiguously darker than the background, and this included all cells from low to high intensities of staining. Thresholds over the background section and the size of the particles were determined by the experimenter. The entire region for each area was traced, and mean densities of c-Fos-immunopositive neurons (number of c-Fos positive cells/mm^2^) for each ROI were calculated according to their respective areas.

### 2.4. Statistical Analysis

Data are presented as means ± standard error of the mean (SEM). Statistical analysis was performed using GraphPad Prism version 5.00 for Windows (GraphPad Software, La Jolla, CA, USA). For the immunohistochemical experiment, the Kolmogorov–Smirnov test revealed parametric (normal) distribution for the c-Fos data for most brain regions. Therefore, differences between groups were assessed using unpaired one-sided *t*-tests. The level of significance was set at *p* < 0.05.

## 3. Results

### 3.1. Lingual LA Stimulation Triggers Neuronal Activation of the Canonical Central Cerebral Gustatory Reward Pathway

**Figure 1:**
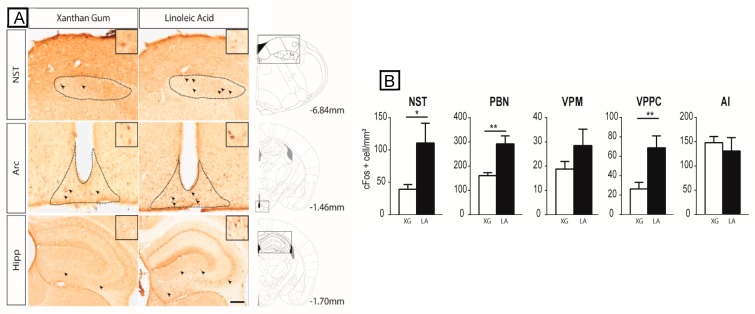
Linoleic acid deposition on the tongue induces c-Fos expression in the major cerebral structures of the canonical gustatory pathway. (**A**) Typical photomicrographs of the NST, Arc, and Hipp, showing c-Fos immunoreactivity in mice subjected to oral stimulation with linoleic acid or xanthan gum (XG, 0.3%, *w*/*v*) to mimic the texture of lipids. The dotted lines circumscribe the regions of interest. Arrowheads point to representative c-Fos immunopositive nuclei. The boxes show higher magnification (×4) of representative c-Fos immunopositive nuclei. Scale bar, 100 µm. The square windows indicate the area shown in the photomicrographs. (**B**) Bar graph representation of the density of c-Fos immunopositive cells (number of c-Fos positive cells/mm^2^) in mice subjected to oral stimulation with linoleic acid (LA) or xanthan gum (XG). Values are means ± standard error of the mean (SEM); *n* = 6; for each structure studied, treatment effects on c-Fos expression were assessed using unpaired one-sided *t*-tests. *, *p* < 0.05; **, *p* < 0.01. AI, agranular insular cortex; Arc, arcuate nucleus; Hipp, hippocampus; NST, nucleus of the solitary tract; PBN, parabrachial nucleus; VPM, ventral posteromedial thalamic nucleus; VPMPC, ventroposterior medialis parvocellularis.

**Figure 2:**
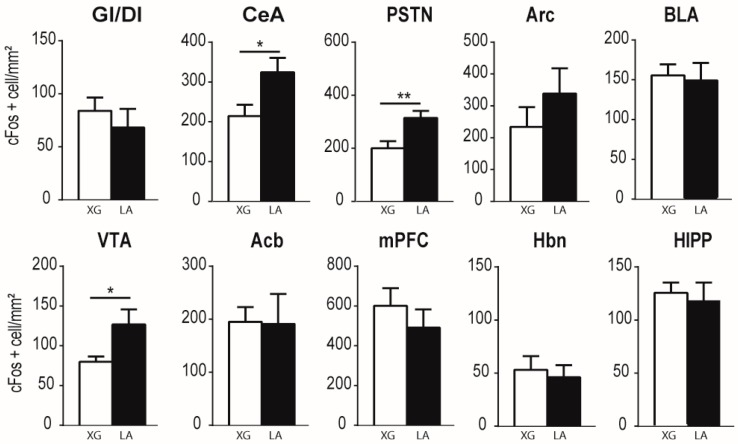
Linoleic acid deposition on the tongue activates c-Fos expression in the major cerebral structures related to emotional and reward traits. Bar graph representation of the density of c-Fos immunopositive cells (number of c-Fos positive cells/mm^2^) in mice subjected to oral stimulation with linoleic acid (LA) or xanthan gum as control solution. Values are means ± SEM; *n* = 6; for each structure studied, treatment effects on c-Fos expression were assessed using unpaired one-sided *t*-tests. * *p* < 0.05; ** *p* < 0.01. Acb, accumbens nucleus; Arc, arcuate nucleus; BLA, basolateral amygdaloid nucleus; CeA, central amygdaloid nucleus; GI/DI, granular/dysgranular insular cortex; Hbn, habenula; Hipp, hippocampus; mPFC, medial prefrontal cortex; PSTN/CbN, parasubthalamic nucleus/calbindin nucleus; VTA, ventral tegmental area.

Figure 1A shows the c-Fos expression in the NST, arcuate nucleus (Arc), and hippocampus (Hipp) in LA-stimulated and control mice. Quantitative analysis of the immunohistochemical data revealed that oral LA application produced a robust increase in c-Fos expression in most brain regions explored along the putative pathway for gustatory lipid perception (Figure 1A,B and Figure 2). More precisely, unpaired one-sided *t*-tests show a significant effect of the treatment in the NST (*t*(10) = 2.252; *p* = 0.0240), PBN (*t*(9) = 3.992; *p* = 0.0016), VPMPC (*t*(9) = 3.153; *p* = 0.0058), CeA (*t*(10) = 2.380; *p* = 0.0193), PSTN/CbN (*t*(9) = 3.037; *p* = 0.0070), and VTA (*t*(10) = 2.309; *p* = 0.0218), but the differences did not reach statistical significance either at the cortical level (AI (*t*(10) = 0.3142; *p* = 0.3799), GI/DI (*t*(10) = 0.05505; *p* = 0.4786), mPFC (*t*(10) = 0.8558; *p* = 0.2061) and HIPP (*t*(9) = 0.3831; *p* = 0.3553)) or in the non-gustatory-related thalamus VPM(*t*(9) = 1.377; *p* = 0.1009), BLA (*t*(10) = 0.2419; *p* = 0.4069), Acb (*t*(10) = 0.06695; *p* = 0.4740), Hbn (*t*(10) = 0.4021; *p* = 0.3480) or Arc (*t*(9) = 1.052; *p* = 0.1602) (Figure 1A,B and Figure 2).

### 3.2. Lingual LA Stimulation Modulates the Expression of mRNA Encoding BDNF, Zif-268 and Glut-1

**Figure 3:**
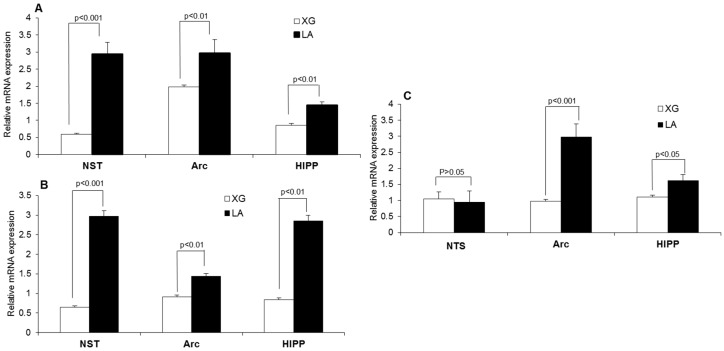
Lingual application of LA modulates brain-derived neurotrophic factor (BDNF), Zif-268, and Glut-1 mRNA expression in the mouse brain. Bar graphs represent the relative increase in mRNA expression (Zif-268 in (**A**), Glut-1 in (**B**), BDNF in (**C**)) in mice subjected to oral stimulation with linoleic acid (LA) or xanthan gum as control solution. Values are means ± SEM; *n* = 6; for each structure studied, treatment effects on Zif-268 and Glut-1 mRNA expression were assessed using unpaired one-sided *t*-tests. NST, nucleus of solitary tract; Arc, arcuate nucleus; Hipp, hippocampus.

The RT-qPCR results show that the relative expression of Zif-268 mRNA was significantly increased (*p* < 0.001) in the NST, arcuate nucleus, and hippocampus by lingual application of LA in mouse brains (Figure 3A). To our surprise, LA induced a nearly fivefold higher increase in Zif-268 mRNA in the NST than controls. LA also resulted in a significantly higher increase (*p* < 0.001) in Glut-1 mRNA expression than control in the nucleus of solitary tract (NST), arcuate nucleus (Arc), and hippocampus (hipp) (Figure 3B). Hence, the increase in Glut-1 mRNA expression was more evident (about a threefold increase) in the NST and hippocampus, as compared to the control solution, after lingual application of LA. The BDNF mRNA expression was increased in the arcuate nucleus and hippocampus, but not in the NTS, after fatty acid lingual application (Figure 3C).

## 4. Discussion

Gene transcription during memory consolidation is a very dynamic and complex process, depending on the type of learning involved. Hence, many types of mRNAs are transcribed, such as for the transcription factors, c-Fos, Zif-268, and the effector genes, like BDNF [20]. The c-Fos, both at mRNA and protein levels, is generally among the first to be expressed and, therefore, referred to as an immediate early gene (IEG) and considered to serve as a marker of the neuronal activity in the neuroendocrine systems [21].

Central gustatory pathways have been well studied in murine models [22]. Branches of the facial (chorda tympani and greater superficial petrosal), glossopharyngeal, and vagus (superior laryngeal) nerves, which establish synaptic contacts with receptor cells in the taste buds, convey taste messages to the first relay nucleus, that is, the rostral part of the nucleus of the solitary tract (NST). The second relay nucleus for ascending taste inputs is the parabrachial nucleus (PBN) of the pons. The third relay station is the ventroposterior medialis parvocellularis (VPMPC) of the thalamus. This thalamic nucleus sends taste information to the insular cortex (IC) [22]. In most of the experiments/observations reported so far, the prominent neurochemical changes in the brain areas take place immediately following training, but in some instances, there are waves from 3 h to 6 h and/or at 24 h following training. In the present study, we were interested in elucidating the early brain responses to short-term application, that is, 30 min, of a long-chain fatty acid, that is, linoleic acid (LA). We applied LA onto the circumvallate papillae as they have been reported to contain more CD36, a lipid receptor, than fungiform papillae [23]. Our results demonstrate that acute lingual application of LA resulted in a significant increase in c-Fos expression in the NST, PBN, and VPMPC in the mouse brain. However, the increase in c-Fos expression in VPM was not significantly higher in fatty-acid-treated mice than control animals, suggesting that VPMPC, but not VPM, is involved in the transfer of lipid taste messages from the PBN to the insular cortical areas (AI, GI/DI), though LA failed to induce c-Fos expression in latter areas of the brain.

The ventral tegmental area (VTA), nucleus accumbens (NAcb), and ventral pallidum (situated between the NAcb and lateral hypothalamus) are the essential components of the brain reward system [24]. Acute application of LA resulted in a significant increase in c-Fos expression in the VTA, which constitutes the mesolimbic dopaminergic system. However, no significant difference in c-Fos expression was observed in the NAcb after the application of the fatty acid. Our observations can be supported by the study of Dela Cruz et al. [25], who have shown that fat intake is associated with the activation of the VTA in the mouse brain [25]. Reward is closely related to hedonic stimuli and our results, showing c-Fos activation in the CEA and PSTN, support previous reports describing the activation of the PSTN, the major target for projection from the CeA [26] in response to hedonic taste [27].

In order to correlate c-Fos findings, we further quantified the mRNA expression of BDNF, Zif-268, and Glut-1 in three areas of the brain, that is, the NTS, arcuate nucleus, and hippocampus, as peripheral signals go through the NTS and arcuate nucleus to the hippocampus, involved in multimodal learning and memory. BDNF is a small dimeric protein that is widely expressed in the adult mammalian brain and extensively involved in synaptic plasticity and memory processes [28]. We observed that BDNF mRNA was highly induced in the arcuate nucleus and hippocampus, but not in the NTS. In fact, this growth factor is more involved in learning and memory rather than the transfer of peripheral signals, as is the case of the NTS. Alonso et al. [29] have demonstrated that BDNF is involved not only in memory consolidation, but also in long-term memory formation in the CA1 region of the hippocampus. Genoud et al. [30] have clearly shown that BDNF is mandatory to induce formation of activity-dependent synapses in cerebral cortex. However, there are regional and task-dependent differences underlying differential mechanisms of BDNF and its receptor function [28].

Zif-268 belongs to the regulatory transcription factor family, responsible for inducing transcription of late-response genes [31]. Induction of memory in the hippocampus has been shown to increase the expression of Zif-268 mRNA, and *Zif-268* knock-out mice had deficits in long-term memory for socially transmitted food preference and object recognition [32]. As far as Glut-1 is concerned, we would like to state that glucose is an important source of energy for the brain and glucose transporters (Glut) enable passage of glucose across both the endothelial cells of the blood–brain barrier and the plasma membranes of neurons and glia [33]. Glut-1 is present at high levels in brain endothelial cells [34]. We observed that lingual application of LA resulted in a significantly increased expression of Zif-268 and Glut-1 mRNA in the NST and arcuate nucleus, as well as in the hippocampus. Surprisingly, the number of c-Fos immunopositive neurons was not significantly increased in the arcuate nucleus and hippocampus by acute application of LA. We would like to mention that, under some conditions, the concomitant activation of these two markers (c-Fos and Zif-268) is not seen. Barbosa et al. [35] have shown that Zif-268 was increased in the dorsal CA1 region of the hippocampus, while there was no c-Fos activation in the experiments conducted to assess episodic-like memory in rats. Furthermore, we elucidated the Zif-268 expression in the whole hippocampus, and it is possible that, in different subregions, there might be a differential expression of Zif-268 mRNA. It is also worth mentioning that c-Fos is well correlated with neuronal activity, whereas Zif-268 is more related to memorization mechanisms, such as long-term potentiation [36,37]. As far as Glut-1 is concerned, we would like to state that sometimes there is no direct correlation between Glut-1 and c-Fos expression. Glut-1 is activated immediately as per energetic demand of the cells, whereas c-Fos might be activated at a later stage. Indeed, Hauguel-de Mouzon et al. [38] have shown that Glut1 mRNA, but not c-Fos levels, is subjected to the variations in glucose concentrations in human placental cells, and this differential regulation of Glut1 and c-Fos genes could be relevant to, respectively, metabolic and mitogenic pathways. In fact, high glucose concentrations are supposed to upregulate c-Fos expression [39] and downregulate Glut-1 levels [36]. It is possible that, in response to lipid gustatory information coming from tongue to brain, the hippocampus is in high requirement of glucose due to high glucose utilization, and this would result in high GLUT-1 and low c-Fos mRNA expression in this region of the brain.

**Figure 4:**
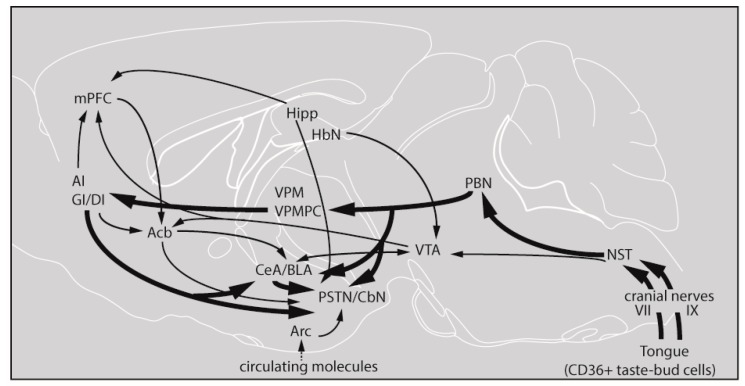
Schematic representation of the gustatory pathway, depicting the major central synaptic relays and their connections with structures involved in metabolic, reward and learning, and memory processes. The lingual application of a long-chain fatty acid will trigger signaling events via CD36, localized in the circumvallate papillae. The gustatory information on dietary lipids will be conveyed to the NST via cranial nerves VII and IX. The NST that serves as the relay structure of the peripheral information will send the gustatory information to different brain areas, as mentioned in the Discussion section. Acb, accumbens nucleus; AI, agranular insular cortex; Arc, arcuate nucleus; BLA, basolateral amygdaloid nucleus; CeA, central amygdaloid nucleus; GI/DI, granular/dysgranular insular cortex; Hbn, habenula; Hipp, hippocampus; mPFC, medial prefrontal cortex; NST, nucleus of the solitary tract; PBN, parabrachial nucleus; PSTN/CbN, parasubthalamic nucleus/calbindin nucleus; VPM, ventral posteromedial thalamic nucleus; VPMPC, ventroposterior medialis parvocellularis; VTA, ventral tegmental area (adapted from Reference [27]).

On the basis of our observations, we provide a schematic representation of the gustatory pathway, depicting the major central synaptic relays and its connections with structures involved in metabolic, reward, learning, and memory processes in response to fatty acid stimulation (Figure 4). Thus, lipid taste perception relies on systematic activation of the major cerebral structures of the canonical gustatory pathway, ranging from the first central synaptic relay NST in the brain stem to the PBN, reaching the gustatory part of the thalamus (VPMPC), up to the gustatory insular cortical areas (AI, GI/DI) with modulatory influences of the central amygdaloid nucleus (CeA) and the posterior part of the lateral hypothalamus, that is, the parasubthalamic nucleus/calbindin nucleus (PSTN/CbN). It is worth noting that, at this early stage of lipid oral stimulation, the reward circuit is already involved, mainly through the VTA. It is not well understood, in the present study, how the arcuate nucleus (Arc), which is sensitive to peripheral postingestive signals, is activated (as evidenced by high BDNF, Zif-268 and Glut-1 mRNA levels). Indeed, the feeding behavior is also regulated by circulating hormonal signals, released by nutrients in the gut, such as cholecystokinin (CCK) and glucagon-like-peptide 1 (GLP-1), released as a result of postingestive/postoral activation of the gastrointestinal tract. Further physiological studies are required to assess the effects of these and other circulating factors that might regulate fat-eating behavior either via the arcuate nucleus or via the vagal nerve X. Nonetheless, ours is the first report to demonstrate that the application of a long-chain fatty acid like linoleic acid would activate a long chain of events in the conscious brain of the mouse.

## References

[1] Hess M.A. (1991). Resetting the American table—Creating a new alliance of taste and health. J. Am. Diet. Assoc..

[2] Chandrashekar J., Hoon M.A., Ryba N.J., Zuker C.S. (2006). The receptors and cells for mammalian taste. Nature.

[3] Roper S.D. (2007). Signal transduction and information processing in mammalian taste buds. Pflugers Arch..

[4] Besnard P., Passilly-Degrace P., Khan N.A. (2016). Taste of fat: A sixth taste modality?. Physiol. Rev..

[5] Ozdener M.H., Subramaniam S., Sundaresan S., Sery O., Hashimoto T., Asakawa Y., Besnard P., Abumrad N.A., Khan N.A. (2014). CD36-and GPR120-mediated Ca^2+^ signaling in human taste bud cells mediates differential responses to fatty acids and is altered in obese mice. Gastroenterology.

[6] Gilbertson T.A., Khan N.A. (2014). Cell signaling mechanisms of oro-gustatory detection of dietary fat: Advances and challenges. Prog. Lipid Res..

[7] Gaillard D., Laugerette F., Darcel N., El-Yassimi A., Passilly-Degrace P., Hichami A., Khan N.A., Montmayeur J.-P., Besnard P. (2008). The gustatory pathway is involved in CD36-mediated orosensory perception of long-chain fatty acids in the mouse. FASEB J..

[8] Kadohisa M., Verhagen J., Rolls E. (2005). The primate amygdala: Neuronal representations of the viscosity, fat texture, temperature, grittiness and taste of foods. Neuroscience.

[9] Verhagen J.V., Rolls E.T., Kadohisa M. (2003). Neurons in the primate orbitofrontal cortex respond to fat texture independently of viscosity. J. Neurophysiol..

[10] Rolls E. (2011). Taste, olfactory and food texture reward processing in the brain and obesity. Int. J. Obes..

[11] Verhagen J.V., Kadohisa M., Rolls E.T. (2004). Primate insular/opercular taste cortex: Neuronal representations of the viscosity, fat texture, grittiness, temperature, and taste of foods. J. Neurophysiol..

[12] Eldeghaidy S., Marciani L., Hort J., Hollowood T., Singh G., Bush D., Foster T., Taylor A.J., Busch J., Spiller R.C. (2016). Prior consumption of a fat meal in healthy adults modulates the brain’s response to fat. J. Nutr..

[13] De Araujo I.E., Rolls E.T. (2004). Representation in the human brain of food texture and oral fat. J. Neurosci..

[14] Eldeghaidy S., Marciani L., McGlone F., Hollowood T., Hort J., Head K., Taylor A.J., Busch J., Spiller R.C., Gowland P.A. (2011). The cortical response to the oral perception of fat emulsions and the effect of taster status. J. Neurophysiol..

[15] Grabenhorst F., Rolls E.T., Parris B.A., d’Souza A.A. (2010). How the brain represents the reward value of fat in the mouth. Cereb. Cortex.

[16] Stice E., Burger K.S., Yokum S. (2013). Relative ability of fat and sugar tastes to activate reward, gustatory, and somatosensory regions. Am. J. Clin. Nutr..

[17] Liu D., Archer N., Duesing K., Hannan G., Keast R. (2016). Mechanism of fat taste perception: Association with diet and obesity. Prog. Lipid Res..

[18] Hichami A., Datiche F., Ullah S., Liénard F., Chardigny J.-M., Cattarelli M., Khan N.A. (2007). Olfactory discrimination ability and brain expression of c-Fos, Gir and Glut1 mRNA are altered in n-3 fatty acid-depleted rats. Behav. Brain Res..

[19] Paxinos G., Franklin K. (2001). The Mouse Brain Atlas in Stereotaxic Coordinates.

[20] Gal-Ben-Ari S., Kenney J.W., Ounalla-Saad H., Taha E., David O., Levitan D., Gildish I., Panja D., Pai B., Wibrand K. (2012). Consolidation and translation regulation. Learn. Mem..

[21] Hoffman G.E., Smith M.S., Verbalis J.G. (1993). C-fos and related immediate early gene products as markers of activity in neuroendocrine systems. Front. Neuroendocrinol..

[22] Morin J.P., Quiroz C., Mendoza-Viveros L., Ramirez-Amaya V., Bermudez-Rattoni F. (2011). Familiar taste induces higher dendritic levels of activity-regulated cytoskeleton-associated protein in the insular cortex than a novel one. Learn. Mem..

[23] Laugerette F., Passilly-Degrace P., Patris B., Niot I., Febbraio M., Montmayeur J.-P., Besnard P. (2005). CD36 involvement in orosensory detection of dietary lipids, spontaneous fat preference, and digestive secretions. J. Clin. Investig..

[24] Wise R.A. (2002). Brain reward circuitry: Insights from unsensed incentives. Neuron.

[25] Dela Cruz J.A., Coke T., Bodnar R.J. (2016). Simultaneous detection of c-fos activation from mesolimbic and mesocortical dopamine reward sites following naive sugar and fat ingestion in rats. J. Vis. Exp..

[26] Chometton S., Pedron S., Peterschmitt Y., Van Waes V., Fellmann D., Risold PY. (2016). A premammillary lateral hypothalamic nuclear complex responds to hedonic but not aversive tastes in the male rat. Brain Struct. Funct..

[27] Barbier M., Chometton S., Peterschmitt Y., Fellmann D., Risold P.Y. (2017). Parasubthalamic and calbindin nuclei in the posterior lateral hypothalamus are the major hypothalamic targets for projections from the central and anterior basomedial nuclei of the amygdala. Brain Struct. Funct..

[28] Murer M.G., Yan Q., Raisman-Vozari R. (2001). Brain-derived neurotrophic factor in the control human brain, and in alzheimer’s disease and parkinson’s disease. Prog. Neurobiol..

[29] Alonso M., Vianna M.R., Depino A.M., Mello e Souza T., Pereira P., Szapiro G., Viola H., Pitossi F., Izquierdo I., Medina J.H. (2002). Bdnf-triggered events in the rat hippocampus are required for both short- and long-term memory formation. Hippocampus.

[30] Genoud C., Knott G.W., Sakata K., Lu B., Welker E. (2004). Altered synapse formation in the adult somatosensory cortex of brain-derived neurotrophic factor heterozygote mice. J. Neurosci..

[31] Lonergan M.E., Gafford G.M., Jarome T.J., Helmstetter F.J. (2010). Time-dependent expression of arc and zif268 after acquisition of fear conditioning. Neural. Plast..

[32] Jones M., Errington M., French P., Fine A., Bliss T., Garel S., Charnay P., Bozon B., Laroche S., Davis S. (2001). A requirement for the immediate early gene zif268 in the expression of late ltp and long-term memories. Nat. Neurosci..

[33] Maher F., Davies-Hill T.M., Lysko P.G., Henneberry R.C., Simpson I.A. (1991). Expression of two glucose transporters, glut1 and glut3, in cultured cerebellar neurons: Evidence for neuron-specific expression of glut3. Mol. Cell Neurosci..

[34] Seidner G., Alvarez M.G., Yeh J.-I., O’Driscoll K.R., Klepper J., Stump T.S., Wang D., Spinner N.B., Birnbaum M.J., Darryl C. (1998). Glut-1 deficiency syndrome caused by haploinsufficiency of the blood-brain barrier hexose carrier. Nat. Genet..

[35] Barbosa F.F., Santos J.R., Meurer Y.S., Macedo P.T., Ferreira L.M., Pontes I.M., Ribeiro A.M., Silva R.H. (2013). Differential cortical c-fos and zif-268 expression after object and spatial memory processing in a standard or episodic-like object recognition task. Front. Behav. Neurosci..

[36] Soares J.G., Pereira A.C., Botelho E.P., Pereira S.S., Fiorani M., Gattass R. (2005). Differential expression of zif268 and c-fos in the primary visual cortex and lateral geniculate nucleus of normal cebus monkeys and after monocular lesions. J. Comp. Neurol..

[37] Aydin-Abidin S., Trippe J., Funke K., Eysel U.T., Benali A. (2008). High-and low-frequency repetitive transcranial magnetic stimulation differentially activates c-fos and zif268 protein expression in the rat brain. Exp. Brain Res..

[38] Hauguel-de Mouzon S., Leturque A., Alsat E., Loizeau M., Evain-Brion D., Girard J. (1994). Developmental expression of glut1 glucose transporter and c-fos genes in human placental cells. Placenta.

[39] Kreisberg J.I., Radnik R.A., Ayo S.H., Garoni J., Saikumar P. (1994). High glucose elevates c-fos and c-jun transcripts and proteins in mesangial cell cultures. Kidney Int..

